# Predicting cigarette use initiation and dependence in adolescence using an affect-driven exploration model

**DOI:** 10.3389/fpsyg.2022.887021

**Published:** 2022-09-05

**Authors:** Atika Khurana, Christopher M. Loan, Dan Romer

**Affiliations:** ^1^Prevention Science Institute, University of Oregon, Eugene, OR, United States; ^2^Department of Educational Methodology, Policy and Leadership, College of Education, University of Oregon, Eugene, OR, United States; ^3^Annenberg Public Policy Center, Annenberg School for Communication, University of Pennsylvania, Philadelphia, PA, United States

**Keywords:** adolescence, affect, risk perception, cigarette use, tobacco use disorder

## Abstract

Adolescent decisions, especially in novel contexts, are often guided by affective evaluations (i.e., feelings associated with a stimulus) rather than knowledge of the risks and probabilities of different outcomes. In this study, we used the affect-driven exploration (ADE) model to illustrate how affective evaluations can play a critical role in driving early use of cigarettes, as well as the adaptive function of the resulting experiential learning in informing future affect and cigarette use. We analyzed five waves of data collected from a large, diverse community sample of adolescents who were followed from early to late adolescence (*N* = 386; 50.9% female; Baseline age = 11.41 ± 0.88 years) during years 2004–2010 to model trajectories of positive affect and risk perceptions (associated with cigarette use) and examined the associations of these trajectories with their self-reported cigarette use and dependence symptoms. Consistent with the ADE model, early initiators reported higher levels of positive affect at baseline, which we argue may have led them to try cigarettes. Notably, most early initiators reported a decline in positive affect over time, suggesting an experience-based shift in affective evaluations associated with cigarette use. Risk perceptions associated with cigarette use did not emerge as a significant predictor of cigarette use or subsequent dependence. Therefore, for deterring adolescent cigarette use, efforts to influence affect (through graphic warning labels and other media) may be more effective than directly influencing risk perceptions. Despite the affective basis for initiating cigarette use, few adolescents engaged in early use (*N* = 20) or developed symptoms of dependence (*N* = 25). Majority of those who engaged in early cigarette use showed a decline in positive affect, with corresponding increase in risk perceptions over time. Some early users may indeed continue to engage in cigarette use, but this is likely driven by the addictive properties of the drug. Overall these findings challenge the popular stereotype of impulsive and emotionally reactive behaviors during adolescence, and suggest a more nuanced interpretation of adolescent risk behavior.

## Introduction

In this study, we test a model that features adolescence as a developmental period in which exploration and experimentation with novel behaviors is necessary for experiential learning and autonomy development. In Western psychology, adolescence has long been characterized as a period of inadequacy, starting with Stanley Hall’s description of adolescence as a period of “storm and stress” (Hall, 1904) to the more recent characterizations, such as “all gas and no brakes” (for a review, see Payne, 2012). Unfortunately, such deficit-oriented depictions of adolescence have done more harm than good to the general understanding of this developmental period which, in fact, is unparalleled in terms of the potential for growth and experiential learning (Romer et al., 2017). By modeling the course of one form of risky behavior in adolescents, namely cigarette use, here we illustrate how adolescent engagement in risk behavior is far from the stereotype of universal storm and stress. We suggest a more nuanced interpretation of adolescent risk behavior, which can serve an adaptive function of promoting experiential learning.

### Affect-driven model of adolescent exploration

When making decisions, especially in uncertain and complex contexts, humans tend to rely on affect i.e., their feelings associated with an object or stimulus (Slovic et al., 2007). The reliance on affect is even more pronounced in adolescent “risky” decision-making, because the context is affectively stimulating (e.g., novel, socially rewarding), complex and uncertain (i.e., outcomes and their probabilities are unknown). A “risky behavior” is typically defined as behavior that has some probability of involving an unfavorable outcome. We argue that the reliance on affect in adolescent “risky” decision-making can in fact be efficient and adaptive if it exposes the adolescent to direct experience of potential consequences (Khurana and Romer, 2022). Affect-guided exploration during adolescence can play a critical role in facilitating experiential learning, and building the affective repertoire of the adolescent for future decision-making. Over time, as adolescents gain experience, their affective tags associated with “risky behaviors” get updated to reflect their actual experiences and associated consequences. They are also likely to acquire more information about behavioral outcomes and their relative probabilities through other sources (e.g., vicarious learning, public health campaigns), which continues to inform their decision-making in later years.

Here, we test the utility of an affect-driven exploration (ADE) model to predict the course of one form of risky behavior in adolescents, namely cigarette use, and show that the reliance on affect-based decision-making in such contexts can be both efficient and adaptive for adolescents, and can promote experiential learning. In the case of cigarette use, the ADE model predicts that adolescents who engage in early cigarette use will be guided by positive affect associated with cigarette use (and not by an evaluation of the risks and benefits associated with smoking). After the initial experimentation, however, adolescents are expected to exhibit a decline in positive affect given their experiences with smoking (e.g., coughing, difficulty breathing, dizziness) and wearing off of the novelty associated with the behavior (Brady et al., 2008). Those who continue using cigarettes will likely do so because their enjoyment of the behavior outweighs its aversiveness and places them at an increased risk for addiction to the drug. By applying the ADE model to predict adolescent cigarette use behaviors, we argue that most adolescents are not necessarily rash and impulsive risk-takers (Payne, 2012), rather they are exploring different choices guided by their affect, and more importantly, they use these experiences to inform future decision-making. Some adolescents are expected to continue smoking and be at risk for dependence as a consequence of the addictive properties of the drug, but these numbers are expected to be far less compared to the universal “storm and stress” portrayals of adolescence.

### Cigarette use in adolescence

Cigarette smoking remains a leading cause of preventable morbidity and mortality in the United States (Bonnie et al., 2015). Despite recent trends of general declines in use and later age of onset (National Institute of Drug Abuse [NIDA], 2020), cigarette smoking still accounts for more than 480,000 deaths each year (Centers for Disease Control and Prevention, 2021) and costs the nation $170 billion annually in health care costs (Xu et al., 2015). Cigarette use onset typically occurs in adolescence, with studies suggesting a link between early use and later dependence (Grant and Dawson, 1998). About 10% of United States eighth graders report having tried a cigarette and that number more than doubles (∼22%) by twelfth grade (National Institute of Drug Abuse [NIDA], 2020). From a prevention standpoint, it is important to understand what factors predict early use and dependence to cigarettes. Here, we examined the role of *affect*, defined as “a positive or negative feeling about an object or stimulus” (Popova et al., 2018), and *perceived risk*, defined as “cognitive evaluations of possible threat or harm” (Sheeran et al., 2014) associated with smoking, as predictors of cigarette use and tobacco use disorder (TUD) symptoms in adolescence.

### Role of affect in predicting smoking behaviors

Slovic et al. (2007) argue that affect can play a crucial role in predicting smoking behaviors, including initiation, continued use, and quitting. Individuals, consciously or unconsciously, tend to be guided by their overall affect (i.e., positive or negative feeling associated with a behavior) when forming judgments about the behavior and deciding whether or not to engage in that behavior. If an individual’s affect toward an object or stimulus is favorable, they tend to perceive lower risks and greater benefits associated with it, and are thus more likely to engage in that behavior (Finucane et al., 2000). Positive affect can therefore lead to reduced perceptions of risks and increased perceptions of benefits associated with the behavior.

The role of affect is especially pertinent in case of adolescents who are biologically wired for seeking out novel behaviors (Spear, 2010), sensitive to peer expectations and approval (Chein et al., 2011), and lacking in experiential knowledge regarding the real consequences associated with cigarette smoking (Romer et al., 2017). Adolescents’ overall affect toward cigarette smoking could also be informed by media depictions of cigarette use, peer or other social norms, as well as for reasons of novelty and adventure (Romer and Hennessy, 2007). While these influences can feed into stereotypes of adolescents as driven by impulses and emotion, it is for these reasons that adolescents are more likely to have positive affect toward cigarette use than adults, which may influence their cigarette use behaviors.

In contrast to the ADE model, which emphasizes reliance on affect in novel and complex decision-making contexts, the analytic or rational model of decision-making argues that individuals weigh the (real or perceived) risks and benefits associated with a behavior when making decisions about whether or not to engage in that behavior. Generally, if the real or perceived risks are greater than the benefits, individuals will choose not to engage in the behavior. Adolescents who report greater perceptions of risk (or harm) associated with cigarette use will be less likely to engage in cigarette use (Song et al., 2009). Other commonly used theoretical frameworks in health psychology (e.g., theory of planned behavior, health belief model) similarly assume human decision-making to be a rational process where individuals make informed decisions weighing the costs and benefits of their actions. However, the ability of humans to act rationally, especially in the context of affective influences, has been questioned by studies across disciplines (e.g., Kahneman, 2003). As such, in the present study we focused on the role of affect in predicting cigarette use behaviors in adolescents, and compared its effects with those of perceived risk.

There is some prior evidence that high levels of perceived risk can deter cigarette use in adolescents (Song et al., 2009), especially among youth who believe that it would be difficult to quit smoking and perceive an earlier onset of health consequences (Gerking and Khaddaria, 2012). Meta-analytic evidence, however, finds weak associations between risk perceptions and involvement in risk behaviors and intentions, especially when evaluated without considering corresponding affect and risk severity (Sheeran et al., 2014). Prior studies have argued that the effect of risk perceptions on adolescent cigarette use onset and progression is likely to be indirect, channeled through overall affect (Romer and Jamieson, 2001). Further, even when adolescents have awareness of the health risks associated with cigarette use, it may not necessarily translate to associating that risk at a personal level, given their lack of direct experiential knowledge. Thus, simply informing adolescents about the risks associated with cigarette use may not be sufficient to deter them from engaging in the behavior, unless this information is communicated in a way that changes their overall affect associated with the behavior. An important case in point is the impact of cigarette warning labels (that elicit strong negative affect) on adolescents’ intentions to use cigarettes (White et al., 2008; Wang et al., 2015) and cigarette use behaviors (Peters et al., 2007; Hammond, 2011).

**FIGURE 1:**
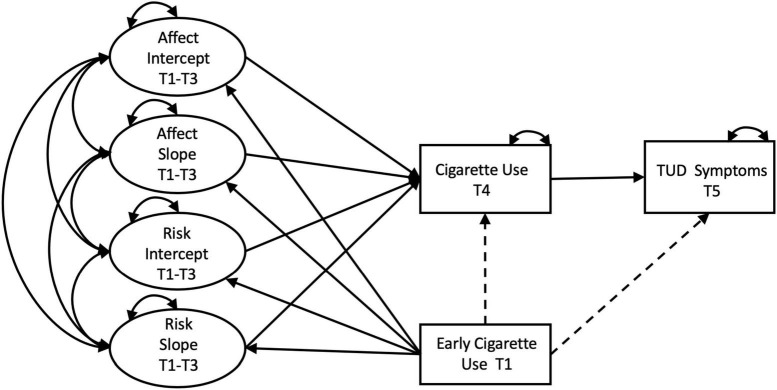
Hypothesized Model. Single-headed straight lines represent regression pathways, double-headed curved lines represent covariances. Dashed pathways signify effects that are hypothesized to be non-significant. Manifest variables corresponding to the latent intercepts and slopes were omitted from the diagram for clarity. Effects of covariates was assessed on intercepts and slopes of affect and risk, and on cigarette use at T4. These pathways have also been omitted for clarity.

In the present study, we analyzed five waves of data (T1–T5) collected from a community-based sample of adolescents to model trajectories of affect and perceived risk from early to middle adolescence (T1–T3), and examined their role as predictors of cigarette use (T4) and dependence (T5) in middle to late adolescence. We further examined how early onset of cigarette use (T1) influences trajectories of affect and perceived risk (T1–T3), and its relation to future cigarette use and dependence. Affect and perceived risk are expected to undergo change during the adolescent years as individuals gain more knowledge about the risks and benefits and experiment with risk behaviors. Nevertheless, prior research has not examined trajectories of affect and risk as predictors of cigarette use. We address this gap and extend current understanding of the role of affect and risk as it relates to early onset of cigarette use, continued use, and dependence across adolescence. Our hypothesized model is presented in Figure 1 and study hypotheses are included below:

*Hypothesis 1 (Trajectories of affect and perceived risk)*: We expected to find significant variance in the latent intercept (i.e., initial values) and slope (i.e., rate of change) factors of affect and perceived risk, with a negative covariance between the intercept factors (Morrell et al., 2010). We expected risk perceptions associated with smoking to increase as adolescents aged due to greater experience with the behavior. Further, the slope of perceived risk was expected to be inversely related with the slope of positive affect, i.e., those who reported more positive affect toward cigarette use were hypothesized to report lower perceived risk associated with smoking (Romer and Jamieson, 2001).

*Hypothesis 2 (Effect of early cigarette use on trajectories of affect and risk):* We hypothesized that early cigarette use would be positively related to the intercept of affect, but negatively associated with the slope of affect. Based on the ADE model, as individuals start smoking, they learn more about the risks and consequences associated with smoking through personal experiences. As such, we expected participants who had already initiated smoking to show a slower increase in affect over time as compared to those who had not initiated smoking. In case of perceived risk, we hypothesized early cigarette use to be negatively associated with the intercept of risk, and positively associated with the slope, such that those who had already initiated smoking would show greater increase in perceived risk over time as compared to those who had not initiated.

*Hypothesis 3 (Relation of affect and risk trajectories and early cigarette use with later cigarette use and TUD in mid-late adolescence):* We expected that the intercept and the slope of affect would be positively associated with cigarette use in mid-adolescence, controlling for the effect of early cigarette use. Further, the intercept and slope of affect were expected to have a significant indirect effect on TUD symptoms (in late adolescence), mediated through cigarette use in mid-adolescence. The effect of intercept and slope of perceived risk on cigarette use in mid-adolescence, and TUD symptoms (in late adolescence) was expected to be weaker in comparison to affect. Finally, we hypothesized that the effect of early cigarette use on later use and dependence would be channeled through trajectories of affect and risk.

## Materials and methods

### Participants

Participants were recruited from schools and community agencies in the Philadelphia area, starting in 2004–2005 and assessed over a period of 8 years, including five annual assessments from 2004 to 2010 (wave 1–5; mean baseline age = 11.41 ± 0.88 years) and a final follow-up after a gap of 2 years (wave 6; mean age = 18.41 ± 0.64 years). The current analyses utilized data from waves 2–6 (i.e., T1–T5). Study participants had a mean age of 12.61 (SD = 0.89; range = 11.0–14.8) years at T1; at T5, participants were 18.8 years old (SD = 0.72; range = 18.0–21.6). Approximately 50.9% (*n* = 189) of the sample reported being female, with majority identifying as non-Hispanic White (55.8%, *n* = 216), followed by non-Hispanic Black (26.4%, *n* = 102), Hispanic (of any race; 9.04%, *n* = 35), and other racial-ethnic groups (8.53%, *n* = 33) including primarily Native American and Asian participants. Average socioeconomic status (SES) measured by the Hollingshead Two-Factor Score was 47.2 on average (SD = 15.39; Range = 15–77). Two-thirds of the sample (66%) was from two-parent households with a median parental education of 14 years. The study was approved by the Institutional Review Board of the Children’s Hospital of Philadelphia. For more details about sample recruitment, see Romer et al. (2009).

### Measures

#### Affect (T1–T3)

Affect toward cigarettes was measured at each wave by asking respondents “*if you smoked a cigarette, even one or two puffs, how would you feel?*” Response options included very bad = 1, somewhat bad = 2, somewhat good = 3, very good = 4. See Table 1 for mean and standard deviations at each wave.

**TABLE 1:** Descriptive statistics and endorsement pattern for affect toward and perceived risk of cigarette use T1–T3.

	Affect					Perceived risk			
	Mean (SD); range	Very bad	Some-what bad	Some-what good	Very good	Mean (SD); range	No	A little	Yes
T1	1.24 (0.52); 1–4	295	64	10	2	2.88 (0.45); 1–3	18	10	343
T2	1.35 (0.67); 1–4	269	68	20	7	2.86 (0.45); 1–3	16	18	330
T3	1.50 (0.76); 1–4	229	87	35	8	2.92 (0.32); 1–3	6	15	338

#### Perceived risk (T1–T3)

Perceived risk associated with cigarette use was measured at each wave by asking participants, “*in your opinion, is cigarette smoking risky for your health?*” Response options included no = 1, a little = 2, yes = 3.

#### Early cigarette use (T1)

Early use was measured dichotomously with the question “*have you ever tried cigarette smoking, even one or two puffs?” (0* = *no, 1* = *yes).* Early use of cigarettes (T1) was endorsed by 20 participants.

#### Cigarette use (T4)

Those who endorsed using cigarettes in their lifetime (yes/no) were asked about *past month* cigarette use on a 7-point scale. To normalize the distribution (skew = 2.46; kurtosis = 4.82) and reduce the number of poorly represented cells (cells with ≤ 5 participants), this was compressed into three response categories: 0 = never tried a cigarette/no lifetime use, 1 = smoked cigarette, but not in past 30 days, 2 = smoked cigarette in past 30 days (skew = 1.59; kurtosis = 0.73). At T4, past month cigarette use was endorsed by 46 participants, while 21 endorsed prior/lifetime use (but not in the past month), and 235 endorsed never trying cigarettes.

#### TUD symptoms (T5)

TUD symptoms were assessed using indicators of abuse and dependence from the 4th edition of the Diagnostic and Statistical Manual of Mental Disorders (DSM-4). Questions pertaining to abuse or dependence were asked only when a participant reported cigarette use in the past year. We matched these indicators of abuse or dependence to the most recent DSM-5 criteria (American Psychiatric Association, 2013). A continuous score was generated which indicated number of criteria met, representing a continuum of severity of disorder. Given high skew (3.87) and kurtosis (15.77), this variable was recoded into three categories (0 = no TUD criteria met; 1 = 1 TUD criterion met, 2 = 2 + TUD criteria met), which substantially lowered skew (2.39) and kurtosis (4.12). At T5, most participants (*n* = 248) met no TUD criteria, 16 met 1 criterion, and 25 met 2 + criteria.

Regarding model covariates, participants self-reported their age, sex (male/female), and race-ethnicity. Family SES was assessed using the Hollingshead Two-Factor Index. Age and family SES were included as continuous covariates, while sex and race-ethnicity were dummy-coded with males and Non-Hispanic Whites coded as the respective reference groups.

### Model specification

To model trajectories of affect and risk, latent growth curves (LGCs) were fit to the data (Bauer and Curran, 2019) using the lavaan 0.6.9 (Rosseel, 2012) package for R (version 4.0.4; R Core Team, 2021). Unconditional LGCs were modeled for affect and perceived risk using T1–T3 data to assess fit, prior to fitting the full model. All indirect effects and 95% confidence intervals (CIs) were estimated with 5,000 bootstrap draws to adjust for bias in the distribution of indirect effects.

Figure 1 shows the full model specification with residuals, demographics, mean structure, and repeated manifest variables omitted for clarity. Intercepts and slopes for both factors were regressed onto age, sex, race-ethnicity, and SES, to assess the conditional structure of both LCGs modeled in parallel to one another; i.e., Parallel Process Model (PPM). Age and SES were coded continuously, while sex (reference group = male) and race/ethnicity (reference group = non-Hispanic white) were dummy coded. In the final model, cigarette use at T4 and TUD symptoms at T5 were regressed on the intercepts and slopes of affect and perceived risk, and early cigarette use at T1. Cigarette use at T4 was also regressed on age, sex, race-ethnicity, and SES to control for their influence. Intercepts and slopes of affect and perceived risk were regressed on early cigarette use at T1 (see Figure 1). Even though early cigarette use and baseline affect and perceived risk were assessed at the same time point, it is reasonable to assume that cigarette use (i.e., have you ever tried smoking) would have happened prior to the T1 assessment, and thus preceded affect and perceived risk reports at T1 in the current data.

The following fit measures were chosen to assess model fit: χ^2^, Robust Comparative Fit Index (Robust CFI), Root Mean Square Error of Approximation (RMSEA), Standardized Root Mean Square Residual (SRMR). According to Hu and Bentler (1999), good model fitting criteria include: CFI ≥ 0.95, RMSEA ≤ 0.06, and SRMR ≤ 0.08. Because χ^2^ significance is inversely dependent on sample size (Bollen, 1986), we did not solely rely on a non-significant χ^2^ test for evaluating model fit.To account for non-normality in the data, we used the robust variant of maximum likelihood (MLR) which outputs Huber-White standard errors and a scaled test statistic that approaches the Yuan-Bentler test statistic asymptotically (Rosseel, 2012). There was 7.33% missingness from T1 to T5, but the data were missing completely at random (MCAR); Little’s MCAR test, χ^2^*(df* = *140)* = 128.35, *p* = 0.75 (Little, 1988). Due to missing exogenous covariates, 16 participants were excluded from this analysis (analysis *N* = 371). Full information maximum likelihood (FIML) was used to account for missing data in all models (Enders, 2010).

## Results

### Unconditional models

Table 1 shows the distribution and mean scores of affect and perceived risk across T1–T3. A linear growth model for affect, χ^2^ (*df* = 1) = 0.62, *p* = 0.43; CFI = 1.00; RMSEA (95% CI) = < 0.01 (<0.01, 0.13), SRMR = < 0.01), perceived risk, χ^2^ (*df* = 1) = 3.82, *p* = 0.05; CFI = 0.98; RMSEA (95% CI) = < 0.01 (< 0.01, 0.09); SRMR = 0.03, and unconditional PPM, χ^2^ (df = 7) = 7.74, *p* = 0.36; CFI = 1.00; RMSEA (95% CI) = 0.02 (<0.01, 0.09); SRMR = 0.03, fit the data well across all metrics. Likelihood ratio tests demonstrated that models with intercept and slope fit the data for affect, χ^2^ *(df* = *3)* = 72.53; *p* < 0.001, and perceived risk, χ^2^ *(df* = *3)* = 14.99; *p* < 0.001, better than their respective intercept-only models. Independent LGCs and PPMs were nearly identical with regard to functional form and estimated parameters, thus only results of PPMs are presented.

Table 2 presents the mean values, and the variance and covariance estimates for the latent intercept and slope factors of affect and perceived risk from the unconditional parallel process model (Table 2). The mean intercept of affect, i.e., the average value of affect for the sample at the first assessment time point, was 1.24 (i.e., between “very bad” and “somewhat bad” in terms of how participant would feel if they smoked a cigarette), the mean slope of affect, i.e., the average rate of change in affect for the sample, was 0.13. There was significant variance (i.e., individual variability around the mean trajectory) in the intercept and slope of affect, but the two factors were not significantly correlated. The mean intercept of perceived risk was 2.87 (between “a little” and “yes” in terms of how risky cigarette smoking is for your health), the mean slope of perceived risk was not significantly different from 0. Significant variance was observed in the intercept value, and near significant variance in the slope of perceived risk (*p* = 0.052). The intercept of perceived risk was negatively correlated with the slope of perceived risk, suggesting that adolescents who had higher risk perceptions at T1 evidenced slower rate of change in risk from T1–T3 as compared to those with lower intercept values. The slope factors of affect and perceived risk were negatively correlated, such that those with higher rates of change in affect had lower rates of change in risk perceptions over time. *R*^2^*s* of manifest variables demonstrated that the linear model explained between 52 and 76% of the variance in affect and between 30 and 76% of the variance in perceived risk across the waves.

**TABLE 2:** Parameter estimates from unconditional parallel process model.

	Unstand. estimate	95% lower CI	95% upper CI	*p*	Standard. estimate
Covariances					
Intercept affect ↔ Slope affect	–0.007	–0.046	0.031	0.709	–0.065
Intercept affect ↔ Intercept risk	–0.006	–0.029	0.018	0.638	–0.035
Intercept affect ↔ Slope risk	–0.011	–0.027	0.005	0.193	–0.143
Slope affect ↔ Intercept risk	–0.002	–0.014	0.010	0.755	–0.018
**Slope affect ↔ Slope risk**	**–0.011**	**–0.021**	**–0.001**	**0.025**	**–0.222**
**Intercept risk ↔ Slope risk**	**–0.065**	**–0.121**	**–0.009**	**0.023**	**–0.898**
Variances					
**Intercept affect**	**0.164**	**0.086**	**0.241**	**0.000**	**1.000**
**Slope affect**	**0.078**	**0.034**	**0.122**	**0.000**	**1.000**
**Intercept risk**	**0.156**	**0.050**	**0.262**	**0.004**	**1.000**
Slope risk	0.033	–0.0003	0.067	0.052	1.000
Mean values					
**Intercept affect**	**1.237**	**2.397**	**3.722**	**<0.001**	**3.060**
**Slope affect**	**0.131**	**0.308**	**0.628**	**<0.001**	**0.468**
**Intercept risk**	**2.872**	**4.737**	**9.800**	**<0.001**	**7.268**
Slope risk	0.023	–0.025	0.275	0.102	0.125

Bold and shaded values signify estimates significant at *p* < 0.05.

### Full model

Table 3 includes regression estimates, and variance and covariance estimates from the full hypothesized model, including the covariates. As predicted, the intercept and slope of affect were significantly and positively related to cigarette use at T4. Further, both intercept and slope of affect had significant, independent, indirect effects on TUD symptoms through cigarette use at T4. The total indirect influence of the intercept and slope of affect on TUD symptoms, i.e., (intercept of affect → cigarette use → TUD symptoms) + (slope of affect → cigarette use → TUD symptoms) was also significant. Table 4 reports the indirect effect estimates from the full model, including (a) total indirect effects of affect on cigarette use at T4 and TUD symptoms at T5, (b) total indirect effects of perceived risk on cigarette use at T4 and TUD symptoms at T5, as well as (c) total indirect effects of early cigarette use (T1) on cigarette use at T4 and TUD symptoms at T5, separated by indirect pathways involving affect and indirect pathways involving perceived risk. In line with our predictions, the effect of intercept and slope of perceived risk on cigarette use and TUD symptoms was weaker than affect. However, contrary to our hypothesis, the effect of intercept and slope of perceived risk on cigarette use outcomes was non-significant.

**TABLE 3:** Parameter estimates from full model.

Direct paths					
***Pathways of influence***	**Unstand. estimate**	**95% Lower CI**	**95% Upper CI**	***p***	**Standard. estimate**
**Intercept affect → Cigarette use (T4)**	**0.698**	**0.424**	**0.971**	**<0.001**	**0.412**
**Slope affect → Cigarette use (T4)**	**1.029**	**0.542**	**1.516**	**<0.001**	**0.417**
Intercept risk → Cigarette use (T4)	–0.120	–0.243	0.004	0.078	–0.065
Slope risk → Cigarette use (T4)	–0.270	–0.846	0.307	0.352	–0.067
Early cigarette use (T1) → Cigarette use (T4)	0.308	–0.288	0.903	0.311	0.096
**T4 cigarette use → TUD symptoms** **(T5)**	**0.492**	**0.345**	**0.638**	**<0.001**	**0.575**
Early cigarette use → TUD symptoms (T5)	0.037	–0.365	0.438	0.858	0.013
**Early cigarette use → Intercept affect**	**0.944**	**0.562**	**1.326**	**0.000**	**0.499**
**Early cigarette use → Slope affect**	**–0.334**	**–0.600**	**–0.067**	**0.014**	**–0.258**
Early cigarette use (T1) → Intercept risk	–0.171	–0.424	0.082	0.186	–0.099
Early cigarette use (T1) → Slope risk	0.106	–0.028	0.241	0.122	0.134
**Covariances**					
Intercept affect ↔ Slope affect	–0.006	–0.043	0.031	0.732	–0.065
Intercept affect ↔ Intercept risk	0.008	–0.011	0.027	0.413	0.058
**Intercept affect ↔ Slope risk**	**–0.017**	**–0.032**	**–0.002**	**0.024**	**–0.274**
Slope affect ↔ Intercept risk	–0.001	–0.013	0.011	0.886	–0.009
**Slope affect ↔ Slope risk**	**–0.011**	**–0.022**	**–0.001**	**0.031**	**–0.235**
**Intercept risk ↔ Slope risk**	**–0.059**	**–0.110**	**–0.008**	**0.023**	**–0.893**
**Variances**					
**Intercept affect**	**0.131**	**0.063**	**0.198**	**0.000**	**0.716**
**Slope affect**	**0.076**	**0.037**	**0.116**	**0.000**	**0.891**
**Intercept risk**	**0.143**	**0.046**	**0.239**	**0.004**	**0.936**
Slope risk	0.030	–0.001	0.062	0.060	0.941

Bold and shaded values signify estimates significant at *p* < 0.05.

**TABLE 4:** Selected indirect effects; 95% confidence intervals (CIs) produced by using the adjusted bootstrap percentile method to adjust for bias in the distribution of indirect effects.

Indirect pathways of influence	Unstan. Est.	95% lower CI	95% upper CI	Stand. Est.
**Indirect effects of affect**				
Intercept affect → Cigarette use (T4) → TUD symptoms (T5)	**0.343**	**0.108**	**0.766**	**0.237**
Slope affect → Cigarette use (T4) → TUD symptoms (T5)	**0.506**	**0.158**	**1.151**	**0.240**
Total indirect effect of affect on cigarette use T4 [(Intercept affect → Cig use T4) + (Slope affect → Cig use T4)]	**1.727**	**1.115**	**2.415**	**0.829**
Total indirect effect of affect on TUD symptoms T5 [(Intercept affect → Cig use T4 → TUD T5) + (Slope affect → Cig use T4→TUD T5)]	**0.849**	**0.504**	**2.415**	**0.477**
**Indirect effects of risk**				
Intercept risk → Cigarette use (T4) → TUD symptoms (T5)	–0.059	–0.173	0.030	–0.037
Slope risk → Cigarette use (T4) → TUD symptoms (T5)	–0.133	–0.607	0.292	–0.039
Total indirect effect of risk on cigarette use T4 [(Intercept risk → Cig use T4) + (Slope risk → Cig Use T4)]	–0.389	–1.327	0.615	–0.132
Total indirect effect of Risk on TUD symptoms T5 [(Intercept risk → Cig use T4→ TUD T5) + (Slope risk → Cig use T4→ TUD T5)]	–0.192	–0.691	0.281	–0.076
**Indirect effects of early cigarette use (T1)**				
Early cigarette use (T1) →Intercept affect → Cigarette use (T4)	**0.659**	**0.152**	**1.373**	**0.206**
Early cigarette use →Slope affect → Cigarette use (T4)	**–0.343**	**–0.974**	**–0.021**	**–0.107**
Total indirect effect of early cigarette use T1 on cigarette use T4 through affect [(Early cigarette use →Intercept affect → Cig use) + (Early cigarette use →Slope affect → Cig use)]	0.315	–0.501	1.233	0.098
Early cigarette use T1 → Intercept affect → Cig use T4 → TUD T5	**0.324**	**0.099**	**0.740**	**0.118**
Early cigarette use T1 → Slope affect → Cig use T4 → TUD T5	**–0.169**	**–0.519**	**–0.015**	**–0.062**
Total indirect effect of early cigarette use T1 on TUD through affect [(Early cigarette use → Intercept affect → Cig use T4 → TUD T5) + (Early cigarette use → Slope affect → Cig use T4 → TUD T5)]	0.155	–0.203	0.675	0.057
Early cigarette use → Intercept risk → Cigarette use T4	0.020	–0.007	0.114	0.006
Early cigarette use →Slope risk → Cigarette use T4	–0.029	–0.210	0.038	–0.009
Total indirect effect of early cigarette use T1 on cigarette use T4 through risk [(Early cigarette use →Intercept risk → Cig use) + (Early cigarette use →Slope risk → Cig use)]	–0.008	–0.155	0.076	–0.003
Early cigarette use T1 → Intercept risk → Cigarette use T4 → TUD T5	0.010	–0.003	0.059	0.004
Early cigarette use T1 → Slope risk → Cigarette use T4 → TUD T5	–0.014	–0.112	0.017	–0.005
Total indirect effect of early cigarette use T1 on TUD T5 through risk [(Early cigarette use → Intercept risk → Cig use T4 → TUD T5) + (Early cigarette use → Slope risk → Cig use T4 → TUD T5)]	–0.004	–0.078	0.037	–0.001

All estimates were created with 5,000 bootstrap draws. Bold and shaded values signify estimates significant at *p* < 0.05.

Early cigarette use (T1) had significant direct effects on the intercept and slope of affect. Early initiators had a higher intercept value but a lower slope value than those who did not initiate early. The effect of early cigarette use (T1) on later cigarette use (at T4) and TUD symptoms (at T5) was indirect, and channeled through both the intercept and slope of affect (see Table 4). Even though the individual indirect effects were significant, given the opposite direction of effects (positive in case of intercept and negative for slope), the total indirect effect of early use through intercept and slope of affect to cigarette use or TUD symptoms was not significant. There was no significant indirect effect of early cigarette use through intercept or slope of perceived risk. Early cigarette use (T1) also did not have any significant direct effects on later cigarette use (T4) or TUD symptoms (T5).

Of the covariates, lower family SES was associated with greater cigarette use at T4, lower intercept of perceived risk, and higher slope of affect (Table A1). Individuals who identified as non-Hispanic Black had lower intercept of perceived risk and lower cigarette use at T4 than the comparison group of non-Hispanic White participants. Participants in the non-Hispanic other racial/ethnic group category had lower slopes of affect and lower cigarette use than non-Hispanic White participants.

**FIGURE 2:**
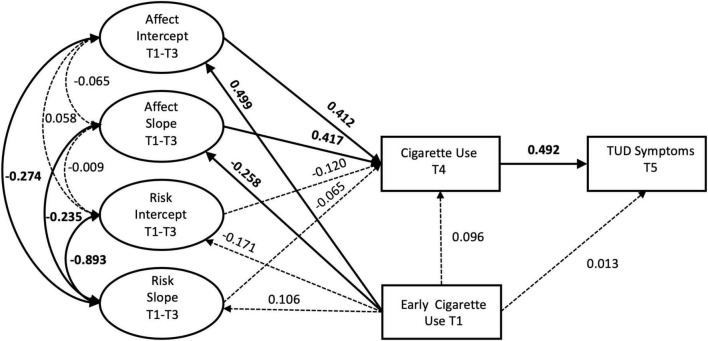
Final model with standardized path estimates. Bolded estimates with solid lines represent significant paths (*p* < 0.05), non-bolded estimates and dotted lines represent non-significant (*p* > 0.05) paths. Covariates, mean structure, and manifest variables for affect and risk not shown.

Overall, our model explained 42% (*R*^2^ = 0.42) and 34% (*R*^2^ = 0.34) of the variance in cigarette use (T4) and TUD symptoms (T5), respectively. Little variance in intercept (*R*^2^ = 0.06) and slope (*R*^2^ = 0.06) of perceived risk was explained by early cigarette use and model covariates, but more variance was explained for intercept (*R*^2^ = 0.28) and slope (*R*^2^ = 0.11) of affect. The full model with all covariates also fit the data well by all metrics, χ^2^ (df = 38) = 46.84, *p* = 0.15; CFI = 0.99; RMSEA (95% CI) = 0.03 (<0.01, 0.05); SRMR = 0.03. Standardized estimates are provided in Figure 2.

## Discussion

In this study, we used the ADE model to examine the role of affect and perceived risk in predicting cigarette use and dependence in adolescents. We modeled trajectories of affect and risk from early to middle adolescence and used these trajectories to predict cigarette use in middle adolescence and TUD symptoms in late adolescence. Further, we accounted for the effect of early cigarette use on the trajectories of affect and risk, and on subsequent cigarette use and TUD symptoms at later waves. Consistent with the ADE model, early initiators reported higher levels of positive affect (associated with cigarette use) at baseline, which we argue may have led them to try cigarettes. Further, affect was not only a stronger predictor of cigarette use in adolescence (as compared to perceived risk), but also a significant predictor of TUD symptoms in late adolescence. Even though perceived risk was negatively associated with affect (Romer and Jamieson, 2001), when accounting for the effect of affect, perceived risk was not a significant predictor of cigarette use or TUD symptoms. Overall, our findings rigorously demonstrate the salience of affect in predicting adolescents’ cigarette use behaviors. Thus, for deterring adolescent cigarette use, efforts to influence affect (through graphic warning labels and other media) may be more effective than directly influencing risk perceptions.

Our findings also have important implications for the “storm and stress” stereotype of adolescence. Despite the important role of affect in guiding adolescent exploration of a risky behavior such as cigarette use, not all adolescents were attracted to such behavior. Furthermore, although early initiators reported high positive affect at baseline, they were not likely to show an increase in affect over time, suggesting an experience-based shift in affective evaluations associated with cigarette use. Only those adolescents who continued to find smoking pleasurable were likely to continue using cigarettes. This of course was likely accentuated by the addictive properties of a drug like nicotine. But even that factor only results in a minority of adolescents exhibiting signs of addiction at the final follow-up. Majority of those who engaged in early cigarette use showed a decline in positive affect, with corresponding increase in risk perceptions over time. Overall our findings challenge the popular stereotype of impulsive and emotionally reactive behaviors during adolescence, and suggest a more nuanced interpretation of adolescent risk behavior.

In terms of prevention programming aimed at reducing cigarette use among adolescents, our findings show that an important mediator of the effect of early cigarette use onset on later dependency is the affect associated with smoking. Both the intercept and slope of positive affect during early adolescence predicted cigarette use at mid-adolescence and subsequent TUD in late adolescence. Further, the lack of a significant association between the intercept and slope of affect suggests that the rate of growth in affect over time is not contingent on baseline levels, which further strengthens the argument that experiential learning can change affect and lead to changes in smoking over time. Since perceived risk was inversely related to positive affect, the significant effects of affect suggest that cognitive evaluations of the health effects of smoking can be over-ridden by the positive affect associated with the behavior. It is important therefore for prevention programs to intervene in this process by demonstrating how smoking can have negative affective consequences. Successful prevention programs can do so by making the consequences of smoking more emotionally evocative (e.g., affective warning labels; Hammond, 2011) and relevant to adolescents (e.g., showing how smoking leads to manipulation and loss of control as exemplified by the Above the Influence Campaign; Carpenter and Pechmann, 2011).

Our findings also show how the levels of affect can change over time in relation to early use. Despite the finding that early cigarette use (at ages 9–11) was positively associated with the intercept of affect, early initiators had significantly lower slopes of affect as compared to those who had not initiated early. This finding reveals that participants who started using cigarettes at an early age showed greater positive affect toward cigarettes (possibly for the rewarding aspect of experimenting with a new behavior); however, over time, this group showed significant declines (rather than an increase) in the positive affect associated with cigarette use. This finding supports the role of affect in guiding early initiation, however, those who initiate early do not necessarily show continued increase in affect long term, possibly due to their declining experience of positive affect associated with smoking. Nevertheless, the overall trajectory of positive affect toward smoking increased with age (from T1 to T3), and this increase predicted greater use of cigarettes at in mid-adolescence and TUD symptoms in late adolescence.

We expected risk perceptions associated with smoking to increase as adolescents aged due to greater experience with the behavior. This increase in perceived risk was hypothesized to be associated with a reduction in positive affect and in smoking (Romer and Hennessy, 2007). Instead, we found a non-significant negative relation between the slope of risk perception and affect. We did, however, find that the early initiators exhibited a decline in positive affect and that this was negatively associated with continued cigarette use as they aged. This finding suggests that as early initiators gain more experiential knowledge associated with cigarette use they become more aware of its deleterious effects.

Perceptions of affect and risk associated with cigarette use have not been modeled together frequently, and no study has modeled trajectories of affect and perceived risk in relation to early use, continued use, and dependence on cigarettes. Romer and Hennessy (2007) examined the effect of risk and affect loaded onto a single latent factor (called affect evaluation). They found that this factor significantly predicted cigarette use in an adolescent sample. However, the comparative magnitudes of the loadings show that affect contributed more strongly to the latent factor than perceived risk. Our study extends these findings by using parallel process modeling, and shows that baseline affect is associated with perceived risk, but accounting for affect, risk does not directly predict cigarette use. In another study with adults, Popova et al. (2018) found that perceived risk predicted cigarette use, but its effect was partially driven by affect, and the magnitude of effect of perceived risk was much lower than that of affect. Our use of latent trajectory modeling enabled us to examine these relationships systematically over time and helped demonstrate the relevance of affect in predicting smoking behaviors during adolescence, consistent with the ADE model. By highlighting the role of affect in “risky” decision-making during adolescence, our findings have important implications for health psychology frameworks that assume that adolescent decisions are based on a rational evaluation of costs and benefits. Despite high levels of perceived risk, adolescent decisions can be biased by their affect associated with the behavior. We also underscore the critical role of experiential learning in informing affect (associated with a behavior) and future decisions to engage in that behavior.

Our findings related to perceived risk are somewhat consistent with prior studies. High and stable levels of perceived risk (associated with cigarette use) are expected during adolescence. For instance, Giovacchini et al. (2017) reported no cross-sectional differences in perceived risk of cigarettes from middle to high school. Song et al. (2009) also reported stable average levels of perceived risk of cigarettes longitudinally over 2 years in high school. By using LGCs, we show the emergence of this high level of perceived risk, such that those with lower starting values have much higher slopes of perceived risk. In fact, the rate of increase was so dramatic that all participants’ predicted trajectories displayed comparable levels of perceived risks by T3, despite different starting values. This demonstrates how risk perceptions become high (and increasingly homogenous) on average among adolescents. We also found that early cigarette use (at T1) was not associated with intercepts or slopes of perceived risk. Further, accounting for the effect of affect, perceived risk did not have a significant influence on cigarette use outcomes in our sample. This makes sense considering that adolescents despite being aware of the perceived risks associated with cigarette use tend to discount the negative impacts it can have at a personal level. The mixed evidence associated with the role of perceived risk as a predictor of smoking behaviors was highlighted in a recent review (Kaufman et al., 2020).

### Limitations and future directions

Our findings are limited in terms of potential self-report bias and issues related to single-item measures for affect and perceived risk. They also may not be generalizable beyond the community sample assessed in this study. Although we interpret the non-significant associations between perceived risk and cigarette use as support for the importance of affect, it is possible that we did not accurately measure risk perceptions and their heterogeneity of effects. It is unclear if our single item assessment of perceived risk was accurately sampling beliefs about risk. Future research should include more sensitive measures of perceived risk.

Future work should also examine potential moderators of the effect of affect and perceived risk on cigarette use, as suggested by theory (e.g., O’Donoghue and Rabin, 2001) and prior research (e.g., Rindfleisch and Crockett, 1999; Krosnick et al., 2006; Kaufman et al., 2020). Interventions to prevent adolescent cigarette use are strongly recommended to closely evaluate heterogeneity in findings before investing in a program that expects risk-based mechanisms to decrease cigarette use. Affect, on the other hand, was clearly shown to be a risk factor for increased smoking and later TUD symptoms. Potential influencers of affect (e.g., peer approval, marketing/advertising, media influences) should be identified and assessed for malleability allowing us to target important predictors of affect toward cigarettes. Given recent trends signaling increased availability and use of electronic nicotine delivery options by adolescents (Cullen et al., 2018), future studies should also assess if the same mechanisms identified in this study underlie attraction to other forms of nicotine, such as e-cigarettes. Finally, because early cigarette use and baseline affect and risk were assessed at the same time point, we are limited in our inference of directionality and can only conclude that adolescents who reported early cigarette use also had higher levels of positive affect at baseline. Future studies using longitudinal data should more rigorously evaluate whether positive affect precedes initial use.

## Data availability statement

The raw data supporting the conclusions of this article will be made available by the authors, without undue reservation.

## Ethics statement

Ethical review and approval was not required for this study in accordance with the local legislation and institutional requirements. Regarding the datasets on which this study was based, the studies involving human participants were reviewed and approved by Institutional Review Board of the Children’s Hospital of Philadelphia. Written informed consent to participate in this study was provided by the participants or their legal guardian/next of kin.

## Author contributions

AK and CL conceived the research questions, conducted the literature review, interpreted results, and helped author all sections of the manuscript. CL conducted the data analysis. AK provided feedback on data analysis and inputs on all sections of the manuscript. DR provided leadership on the overarching study and feedback on all sections of the manuscript. All authors have read and approved the final manuscript.

## Appendix

**TABLE A1:** Demographic parameter estimates from full model.

Direct paths						
**Outcome**	**Predictor**	**Unstand. estimate**	**95% lower CI**	**95% upper CI**	***p***	**Standard. estimate**
Cigarette use (T4)	Sex (Female)	0.001	–0.133	0.134	0.994	<0.001
	Non-Hispanic Black	**–0.215**	**–0.386**	**–0.044**	**0.012**	**–0.132**
	Hispanic	0.238	–0.061	0.537	0.118	0.094
	**Other race/ethnicity**	**–0.224**	**–0.356**	**–0.092**	**0.001**	**–0.088**
	**SES**	**–0.005**	**–0.009**	**–0.001**	**0.020**	**–0.102**
Intercept affect	Age	0.037	–0.015	0.088	0.160	0.076
	Sex (Female)	–0.021	–0.114	0.073	0.663	–0.024
	Non-Hispanic Black	0.107	–0.014	0.227	0.083	0.110
	Hispanic	–0.021	–0.184	0.142	0.802	–0.014
	Other race/ethnicity	–0.037	–0.159	0.085	0.553	–0.025
	SES	0.000	–0.003	0.003	0.964	0.002
Slope affect	Age	0.038	–0.003	0.079	0.071	0.115
	Sex (Female)	–0.024	–0.100	0.051	0.527	–0.041
	Non-Hispanic Black	–0.005	–0.102	0.092	0.918	–0.008
	Hispanic	0.002	–0.155	0.160	0.978	0.002
	**Other race/ethnicity**	**–0.110**	**–0.212**	**–0.009**	**0.033**	**–0.107**
	**SES**	**–0.003**	**–0.005**	**–0.001**	**0.003**	**–0.168**
Intercept risk	Age	–0.013	–0.065	0.038	0.611	–0.030
	Sex (Female)	0.039	–0.055	0.133	0.417	0.050
	**Non-Hispanic Black**	**–0.150**	**–0.284**	**–0.017**	**0.027**	**–0.170**
	Hispanic	0.051	–0.079	0.182	0.440	0.037
	Other race/ethnicity	0.014	–0.105	0.134	0.814	0.010
	**SES**	**0.003**	**0.000**	**0.006**	**0.048**	**0.119**
Slope risk	Age	0.006	–0.021	0.033	0.661	0.030
	Sex (Female)	–0.036	–0.088	0.017	0.184	–0.099
	Non-Hispanic Black	0.071	–0.004	0.147	0.064	0.175
	Hispanic	–0.014	–0.099	0.071	0.745	–0.022
	Other race/ethnicity	0.021	–0.053	0.095	0.581	0.033
	SES	–0.001	–0.002	0.001	0.434	–0.054

Bold values signify estimates significant at *p* < 0.05.
